# (1-Adamant­yl){4-[(2-chloro-9-isopropyl-9*H*-purin-6-yl)aminometh­yl]phen­yl}methanone trichloro­methane solvate

**DOI:** 10.1107/S1600536809016596

**Published:** 2009-05-14

**Authors:** Michal Rouchal, Marek Nečas, Robert Vícha

**Affiliations:** a Department of Chemistry, Faculty of Technology, Tomas Bata University in Zlin, Nám. T. G. Masaryka 275, Zlín 762 72, Czech Republic; b Department of Chemistry, Faculty of Science, Masaryk University in Brno, Kamenice 5, Brno-Bohunice 625 00, Czech Republic

## Abstract

In the title compound, C_26_H_30_ClN_5_O·CHCl_3_, the purine mol­ecule consists of essentially planar benzene and purine ring systems [maximum deviation 0.010 (4) Å for both ring systems] forming a dihedral angle of 85.52 (9)°. Inter­molecular N—H⋯N hydrogen bonds link adjacent mol­ecules into centrosymmetric dimers. The structure also contains inter­molecular C—H⋯O and C—H⋯N inter­actions. The benzene rings form offset face-to-face π–π stacking inter­actions with an inter­planar distance of 3.541 (4) Å and a centroid-to-centroid distance of 4.022 (4) Å.

## Related literature

The title compound was prepared according to a modified literature procedure (Fiorini & Abel, 1998 ▶). For the synthesis and/or biological activity of related compounds, see: Legraverend & Grierson (2006 ▶); Long *et al.* (2007 ▶). For related structures, see: Trávníček & Kryštof (2004 ▶); Trávníček & Zatloukal (2004 ▶); Trávníček & Popa (2007*a* ▶,*b* ▶) Rouchal *et al.* (2009 ▶).
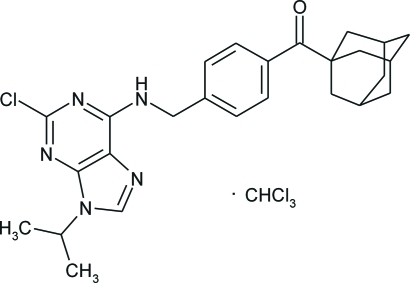


## Experimental

### 

#### Crystal data

C_26_H_30_ClN_5_O·CHCl_3_
*M* *_r_* = 583.39
Orthorhombic, 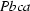

*a* = 19.434 (12) Å
*b* = 13.186 (7) Å
*c* = 22.149 (11) Å
*V* = 5676 (5) Å^3^
*Z* = 8
Mo *K*α radiation
μ = 0.45 mm^−1^
*T* = 120 K
0.45 × 0.40 × 0.20 mm

#### Data collection

Kuma KM4 CCD diffractometer
Absorption correction: multi-scan (*CrysAlis RED*; Oxford Diffraction, 2006 ▶) *T*_min_ = 0.738, *T*_max_ = 0.917
32219 measured reflections
4984 independent reflections
2981 reflections with *I* > 2σ(*I*)
*R*_int_ = 0.044

#### Refinement

*R*[*F* ^2^ > 2σ(*F* ^2^)] = 0.050
*wR*(*F* ^2^) = 0.177
*S* = 1.12
4984 reflections
334 parameters
H-atom parameters constrained
Δρ_max_ = 0.71 e Å^−3^
Δρ_min_ = −0.54 e Å^−3^

### 

Data collection: *CrysAlis CCD* (Oxford Diffraction, 2006 ▶); cell refinement: *CrysAlis RED* (Oxford Diffraction, 2006 ▶); data reduction: *CrysAlis RED*; program(s) used to solve structure: *SHELXS97* (Sheldrick, 2008 ▶); program(s) used to refine structure: *SHELXL97* (Sheldrick, 2008 ▶); molecular graphics: *ORTEP-3* (Farrugia, 1997 ▶); software used to prepare material for publication: *SHELXL97*.

## Supplementary Material

Crystal structure: contains datablocks global, I. DOI: 10.1107/S1600536809016596/bi2361sup1.cif

Structure factors: contains datablocks I. DOI: 10.1107/S1600536809016596/bi2361Isup2.hkl

Additional supplementary materials:  crystallographic information; 3D view; checkCIF report

## Figures and Tables

**Table 1:** Hydrogen-bond geometry (Å, °)

*D*—H⋯*A*	*D*—H	H⋯*A*	*D*⋯*A*	*D*—H⋯*A*
N1—H1*A*⋯N5^i^	0.88	2.22	3.013 (5)	150
C27—H27*A*⋯N2^ii^	1.00	2.59	3.553 (6)	161
C5—H5*B*⋯N3^iii^	1.00	2.66	3.641 (5)	166
C23—H23*A*⋯O1^iv^	0.95	2.23	3.179 (5)	175

Symmetry codes: (i) 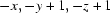; (ii) 
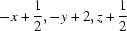
; (iii) 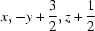; (iv) 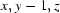.

**Fig. 1.**
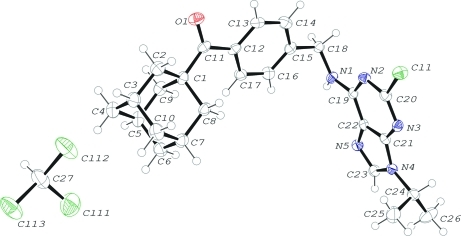
The molecular structure with displacement ellipsoids drawn at 50% probability for non-H atoms.

**Fig. 2.**
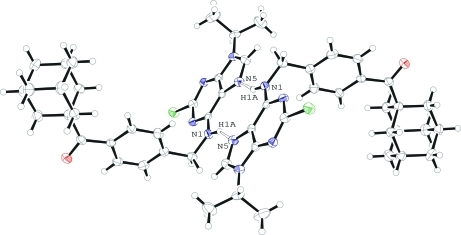
Centrosymmetric dimer linked by N—H···N hydrogen bonds (dashed lines). Displacement ellipsoids are drawn at 50% probability for non-H atoms.

## supplementary crystallographic information

### Comment

Purine molecules substituted at C2, C6 and N9 are known as potential ATP-competitive inhibitors of cyclin-dependent kinases (key regulators of the cell division cycle) and due to this fact, they can show an anticancer activity (Legraverend & Grierson, 2006). The adamantane scaffold is often used in order to improve the biological properties of potential or known drugs and a number of compounds with various biological activity have been described, *e.g.* antitumor agents published by Long *et al.* (2007).

The asymmetric unit of the title compound (Fig. 1) consists of a trisubstituted purine molecule with trichloromethane solvent in the ratio of one to one. Both benzene and purine rings are essentially planar with maximum deviation from the best plane being 0.010 (4) Å for C19 of the purine ring and 0.010 (4) Å for C16 of the benzene ring. The dihedral angle between purine and benzene rings is 85.52 (9)°. The torsion angles C18/N1/C19/C22, C19/N1/C18/C15, N1/C18/C15/C16 and H24A/C24/N4/C21 are 173.42 (4), -79.52 (4), -36.80 (5) and -34.28 (5)° respectively. Adjacent molecules are linked into centrosymmetric pairs by intermolecular N1—H1A···N5^i^ hydrogen bonds (Table 2, Fig. 2; symmetry code: (i) -x, 1 - y, 1 - z). The trichloromethane molecule forms intermolecular C—H···N and C—H···Cl interactions. Additional intermolecular interactions (Table 2) include C23—H23A···O1, C5—H5B···N3, C—H···Cl contacts and offset face-to-face π–π interactions with an interplanar distance of 3.541 (4) Å and a centroid–centroid distance of 4.022 (4) Å.

### Experimental

The title compound was prepared according to a slightly modified literature procedure (Fiorini & Abel, 1998). 2,6-Dichloro-9-(propan-2-yl)-9*H*-purine (0.53 mmol, 122.5 mg) and (1-adamantyl)-[4-(aminomethyl)phenyl]methanone hydrochloride (0.56 mmol, 170 mg) were dissolved in a mixture of DMF (2 ml) and triethylamine (1.06 mmol, 0.15 ml). The resulting solution was stirred under reflux for 2 h (the reaction progress was monitored by TLC). After this period, the mixture was diluted with water and extracted five times with 15 ml of diethyl ether. The combined organic layers were washed twice with brine and dried over sodium sulfate. The desired product was obtained by evaporation of the solvent in vacuum and purified by column chromatography (silica gel; petroleum ether/ethyl acetate, *v*/*v* 1:1) to give a colourless crystalline powder (210 mg, 86%, mp 190–192°C). The crystal used for data collection was acquired by evaporation from a chloroform solution at room temperature.

### Crystal data

**Table:**

C_26_H_30_ClN_5_O·CHCl_3_	*D*_x_ = 1.365 Mg m^−^^3^
*M**_r_* = 583.39	Melting point: 191 K
Orthorhombic, *P**b**c**a*	Mo *K*α radiation, λ = 0.71073 Å
Hall symbol: -P 2ac 2ab	Cell parameters from 4984 reflections
*a* = 19.434 (12) Å	θ = 2.6–25.0°
*b* = 13.186 (7) Å	µ = 0.45 mm^−^^1^
*c* = 22.149 (11) Å	*T* = 120 K
*V* = 5676 (5) Å^3^	Block, colourless
*Z* = 8	0.45 × 0.40 × 0.20 mm
*F*(000) = 2432	

### Data collection

**Table:**

Kuma KM4 CCD diffractometer	4984 independent reflections
Radiation source: fine-focus sealed tube	2981 reflections with *I* > 2σ(*I*)
graphite	*R*_int_ = 0.044
Detector resolution: 0.06 pixels mm^-1^	θ_max_ = 25.0°, θ_min_ = 2.6°
ω scans	*h* = −23→21
Absorption correction: multi-scan (*CrysAlis RED*; Oxford Diffraction, 2006)	*k* = −15→15
*T*_min_ = 0.738, *T*_max_ = 0.917	*l* = −26→21
32219 measured reflections	

### Refinement

**Table:**

Refinement on *F*^2^	Primary atom site location: structure-invariant direct methods
Least-squares matrix: full	Secondary atom site location: difference Fourier map
*R*[*F*^2^ > 2σ(*F*^2^)] = 0.050	Hydrogen site location: inferred from neighbouring sites
*wR*(*F*^2^) = 0.177	H-atom parameters constrained
*S* = 1.12	*w* = 1/[σ^2^(*F*_o_^2^) + (0.0729*P*)^2^ + 9.6676*P*] where *P* = (*F*_o_^2^ + 2*F*_c_^2^)/3
4984 reflections	(Δ/σ)_max_ < 0.001
334 parameters	Δρ_max_ = 0.71 e Å^−^^3^
0 restraints	Δρ_min_ = −0.54 e Å^−^^3^

### Special details

**Table:**

Geometry. All e.s.d.'s (except the e.s.d. in the dihedral angle between two l.s. planes) are estimated using the full covariance matrix. The cell e.s.d.'s are taken into account individually in the estimation of e.s.d.'s in distances, angles and torsion angles; correlations between e.s.d.'s in cell parameters are only used when they are defined by crystal symmetry. An approximate (isotropic) treatment of cell e.s.d.'s is used for estimating e.s.d.'s involving l.s. planes.
Refinement. Refinement of *F*^2^ against ALL reflections. The weighted *R*-factor *wR* and goodness of fit *S* are based on *F*^2^, conventional *R*-factors *R* are based on *F*, with *F* set to zero for negative *F*^2^. The threshold expression of *F*^2^ > 2σ(*F*^2^) is used only for calculating *R*-factors(gt) *etc*. and is not relevant to the choice of reflections for refinement. *R*-factors based on *F*^2^ are statistically about twice as large as those based on *F*, and *R*- factors based on ALL data will be even larger.

### Fractional atomic coordinates and isotropic or equivalent isotropic displacement parameters (Å^2^)

**Table:**

	*x*	*y*	*z*	*U*_iso_*/*U*_eq_	
Cl1	0.15090 (5)	0.68048 (7)	0.25476 (4)	0.0305 (3)	
O1	0.13811 (13)	1.1399 (2)	0.50691 (13)	0.0320 (7)	
N1	0.00385 (14)	0.6307 (2)	0.43338 (14)	0.0230 (7)	
H1A	−0.0135	0.5931	0.4625	0.028*	
N2	0.07497 (15)	0.6451 (2)	0.34920 (14)	0.0226 (7)	
N3	0.15847 (14)	0.5170 (2)	0.32042 (13)	0.0217 (7)	
N4	0.15553 (15)	0.3704 (2)	0.38744 (13)	0.0233 (7)	
N5	0.07206 (15)	0.4204 (2)	0.45195 (14)	0.0256 (7)	
C1	0.21373 (18)	1.0428 (3)	0.56796 (17)	0.0254 (9)	
C2	0.2555 (2)	1.1416 (3)	0.56949 (19)	0.0307 (9)	
H2B	0.2251	1.1982	0.5819	0.037*	
H2C	0.2729	1.1566	0.5284	0.037*	
C3	0.3165 (2)	1.1347 (3)	0.61349 (18)	0.0322 (10)	
H3B	0.3428	1.2000	0.6132	0.039*	
C4	0.2899 (2)	1.1132 (3)	0.67731 (19)	0.0355 (10)	
H4A	0.2600	1.1696	0.6908	0.043*	
H4B	0.3292	1.1080	0.7056	0.043*	
C5	0.2487 (2)	1.0128 (3)	0.67746 (18)	0.0322 (10)	
H5B	0.2315	0.9988	0.7192	0.039*	
C6	0.2956 (2)	0.9260 (3)	0.65710 (19)	0.0361 (10)	
H6A	0.2695	0.8615	0.6572	0.043*	
H6B	0.3346	0.9192	0.6856	0.043*	
C7	0.3232 (2)	0.9470 (3)	0.59318 (18)	0.0299 (9)	
H7A	0.3539	0.8901	0.5805	0.036*	
C8	0.26304 (19)	0.9563 (3)	0.54841 (17)	0.0270 (9)	
H8A	0.2375	0.8914	0.5469	0.032*	
H8B	0.2811	0.9705	0.5075	0.032*	
C9	0.18688 (19)	1.0227 (3)	0.63391 (17)	0.0282 (9)	
H9A	0.1570	1.0794	0.6469	0.034*	
H9B	0.1593	0.9596	0.6347	0.034*	
C10	0.3642 (2)	1.0465 (3)	0.59343 (19)	0.0340 (10)	
H10A	0.3824	1.0603	0.5525	0.041*	
H10B	0.4036	1.0408	0.6216	0.041*	
C11	0.15273 (18)	1.0551 (3)	0.52472 (17)	0.0239 (9)	
C12	0.10965 (18)	0.9675 (3)	0.50235 (17)	0.0243 (9)	
C13	0.07214 (18)	0.9841 (3)	0.44859 (17)	0.0271 (9)	
H13A	0.0749	1.0479	0.4288	0.033*	
C14	0.03137 (19)	0.9082 (3)	0.42450 (18)	0.0300 (9)	
H14A	0.0066	0.9209	0.3883	0.036*	
C15	0.02571 (18)	0.8139 (3)	0.45191 (17)	0.0241 (8)	
C16	0.06142 (19)	0.7977 (3)	0.50545 (18)	0.0282 (9)	
H16A	0.0571	0.7345	0.5256	0.034*	
C17	0.10368 (19)	0.8731 (3)	0.53013 (18)	0.0266 (9)	
H17A	0.1285	0.8598	0.5662	0.032*	
C18	−0.02097 (19)	0.7331 (3)	0.42573 (17)	0.0248 (9)	
H18A	−0.0668	0.7388	0.4450	0.030*	
H18B	−0.0270	0.7465	0.3821	0.030*	
C19	0.05284 (17)	0.5906 (3)	0.39739 (16)	0.0206 (8)	
C20	0.12500 (18)	0.6035 (3)	0.31567 (16)	0.0242 (9)	
C21	0.13385 (17)	0.4650 (3)	0.36843 (16)	0.0213 (8)	
C22	0.08232 (17)	0.4945 (3)	0.40833 (16)	0.0203 (8)	
C23	0.11642 (19)	0.3486 (3)	0.43759 (17)	0.0251 (9)	
H23A	0.1209	0.2874	0.4599	0.030*	
C24	0.21277 (18)	0.3110 (3)	0.35970 (18)	0.0279 (9)	
H24A	0.2129	0.3250	0.3153	0.033*	
C25	0.2812 (2)	0.3476 (4)	0.3856 (2)	0.0502 (13)	
H25A	0.2864	0.4203	0.3779	0.075*	
H25B	0.3191	0.3106	0.3664	0.075*	
H25C	0.2821	0.3352	0.4292	0.075*	
C26	0.2007 (2)	0.1985 (3)	0.3687 (2)	0.0460 (12)	
H26A	0.1564	0.1796	0.3508	0.069*	
H26B	0.2001	0.1831	0.4120	0.069*	
H26C	0.2377	0.1602	0.3491	0.069*	
C27	0.5087 (2)	1.1979 (4)	0.7415 (2)	0.0520 (14)	
H27A	0.4751	1.2334	0.7683	0.062*	
Cl11	0.49438 (8)	1.06661 (13)	0.74624 (7)	0.0720 (5)	
Cl12	0.49582 (6)	1.23926 (13)	0.66603 (5)	0.0630 (4)	
Cl13	0.59279 (6)	1.22798 (13)	0.76534 (6)	0.0635 (5)	

### Atomic displacement parameters (Å^2^)

**Table:**

	*U*^11^	*U*^22^	*U*^33^	*U*^12^	*U*^13^	*U*^23^
Cl1	0.0316 (5)	0.0309 (6)	0.0291 (5)	0.0028 (4)	0.0083 (4)	0.0085 (4)
O1	0.0316 (15)	0.0228 (15)	0.0416 (18)	0.0013 (12)	−0.0007 (12)	0.0059 (13)
N1	0.0249 (16)	0.0199 (17)	0.0242 (17)	−0.0002 (13)	0.0038 (13)	0.0003 (14)
N2	0.0203 (15)	0.0249 (17)	0.0224 (17)	0.0011 (13)	0.0007 (13)	0.0029 (14)
N3	0.0206 (15)	0.0228 (17)	0.0216 (17)	0.0001 (13)	0.0018 (13)	−0.0025 (14)
N4	0.0219 (16)	0.0286 (19)	0.0194 (17)	0.0041 (13)	0.0006 (13)	−0.0001 (14)
N5	0.0247 (16)	0.0278 (19)	0.0243 (18)	0.0027 (14)	−0.0008 (14)	−0.0014 (15)
C1	0.025 (2)	0.025 (2)	0.026 (2)	0.0004 (16)	0.0031 (16)	−0.0034 (17)
C2	0.033 (2)	0.026 (2)	0.033 (2)	−0.0035 (18)	0.0026 (18)	0.0040 (18)
C3	0.032 (2)	0.029 (2)	0.035 (2)	−0.0052 (18)	−0.0028 (18)	−0.0020 (19)
C4	0.040 (2)	0.035 (3)	0.031 (2)	−0.0030 (19)	0.0001 (19)	−0.0044 (19)
C5	0.038 (2)	0.031 (2)	0.027 (2)	−0.0051 (19)	0.0010 (18)	−0.0044 (19)
C6	0.041 (2)	0.032 (2)	0.035 (3)	−0.0031 (19)	−0.0089 (19)	0.000 (2)
C7	0.026 (2)	0.030 (2)	0.034 (2)	0.0021 (17)	0.0001 (17)	−0.0043 (18)
C8	0.027 (2)	0.027 (2)	0.027 (2)	0.0045 (17)	0.0045 (16)	−0.0048 (17)
C9	0.029 (2)	0.027 (2)	0.029 (2)	−0.0016 (17)	0.0082 (17)	−0.0007 (18)
C10	0.025 (2)	0.043 (3)	0.034 (2)	−0.0083 (19)	0.0008 (18)	−0.003 (2)
C11	0.0235 (19)	0.021 (2)	0.028 (2)	0.0037 (16)	0.0087 (16)	0.0004 (17)
C12	0.0178 (18)	0.021 (2)	0.034 (2)	0.0045 (15)	0.0049 (16)	−0.0014 (18)
C13	0.0251 (19)	0.026 (2)	0.030 (2)	0.0045 (17)	0.0018 (17)	0.0063 (18)
C14	0.025 (2)	0.037 (3)	0.029 (2)	0.0029 (18)	−0.0020 (17)	0.0032 (19)
C15	0.0221 (19)	0.025 (2)	0.025 (2)	0.0013 (16)	0.0078 (15)	−0.0009 (17)
C16	0.029 (2)	0.029 (2)	0.027 (2)	−0.0014 (17)	0.0030 (17)	0.0037 (18)
C17	0.029 (2)	0.022 (2)	0.029 (2)	−0.0009 (16)	−0.0039 (17)	0.0028 (17)
C18	0.0267 (19)	0.025 (2)	0.023 (2)	0.0044 (16)	0.0016 (16)	0.0000 (17)
C19	0.0153 (17)	0.021 (2)	0.025 (2)	−0.0035 (15)	−0.0049 (15)	−0.0059 (17)
C20	0.0238 (19)	0.028 (2)	0.021 (2)	0.0000 (16)	0.0008 (16)	0.0018 (17)
C21	0.0197 (18)	0.025 (2)	0.020 (2)	−0.0020 (15)	−0.0032 (15)	−0.0002 (17)
C22	0.0219 (18)	0.021 (2)	0.0178 (19)	−0.0035 (15)	−0.0017 (14)	−0.0018 (16)
C23	0.030 (2)	0.026 (2)	0.020 (2)	0.0019 (16)	0.0021 (16)	0.0025 (17)
C24	0.0207 (19)	0.037 (2)	0.026 (2)	0.0063 (17)	0.0015 (16)	−0.0011 (18)
C25	0.028 (2)	0.070 (4)	0.053 (3)	0.012 (2)	−0.006 (2)	−0.027 (3)
C26	0.048 (3)	0.036 (3)	0.054 (3)	0.011 (2)	0.023 (2)	0.007 (2)
C27	0.035 (2)	0.087 (4)	0.034 (3)	0.009 (3)	−0.003 (2)	−0.013 (3)
Cl11	0.0769 (10)	0.0811 (11)	0.0581 (9)	0.0080 (8)	0.0082 (7)	−0.0164 (8)
Cl12	0.0379 (7)	0.1163 (13)	0.0348 (7)	0.0103 (7)	−0.0040 (5)	−0.0052 (7)
Cl13	0.0324 (6)	0.1206 (13)	0.0377 (7)	0.0056 (7)	−0.0026 (5)	−0.0095 (7)

### Geometric parameters (Å, °)

**Table:**

Cl1—C20	1.762 (4)	C8—H8A	0.990
O1—C11	1.219 (4)	C8—H8B	0.990
N1—C19	1.349 (5)	C9—H9A	0.990
N1—C18	1.445 (5)	C9—H9B	0.990
N1—H1A	0.880	C10—H10A	0.990
N2—C20	1.341 (5)	C10—H10B	0.990
N2—C19	1.357 (5)	C11—C12	1.511 (5)
N3—C20	1.316 (5)	C12—C17	1.392 (5)
N3—C21	1.353 (5)	C12—C13	1.413 (5)
N4—C23	1.376 (5)	C13—C14	1.384 (5)
N4—C21	1.383 (5)	C13—H13A	0.950
N4—C24	1.493 (5)	C14—C15	1.388 (5)
N5—C23	1.320 (5)	C14—H14A	0.950
N5—C22	1.388 (5)	C15—C16	1.390 (5)
C1—C11	1.532 (5)	C15—C18	1.515 (5)
C1—C2	1.535 (5)	C16—C17	1.401 (5)
C1—C8	1.552 (5)	C16—H16A	0.950
C1—C9	1.574 (5)	C17—H17A	0.950
C2—C3	1.538 (6)	C18—H18A	0.990
C2—H2B	0.990	C18—H18B	0.990
C2—H2C	0.990	C19—C22	1.411 (5)
C3—C4	1.531 (6)	C21—C22	1.391 (5)
C3—C10	1.551 (6)	C23—H23A	0.950
C3—H3B	1.000	C24—C26	1.515 (6)
C4—C5	1.548 (6)	C24—C25	1.526 (6)
C4—H4A	0.990	C24—H24A	1.000
C4—H4B	0.990	C25—H25A	0.980
C5—C6	1.531 (6)	C25—H25B	0.980
C5—C9	1.546 (6)	C25—H25C	0.980
C5—H5B	1.000	C26—H26A	0.980
C6—C7	1.539 (6)	C26—H26B	0.980
C6—H6A	0.990	C26—H26C	0.980
C6—H6B	0.990	C27—Cl11	1.756 (6)
C7—C10	1.535 (6)	C27—Cl13	1.763 (5)
C7—C8	1.537 (5)	C27—Cl12	1.775 (5)
C7—H7A	1.000	C27—H27A	1.000
			
C19—N1—C18	122.2 (3)	H10A—C10—H10B	108.2
C19—N1—H1A	118.9	O1—C11—C12	117.8 (3)
C18—N1—H1A	118.9	O1—C11—C1	118.7 (3)
C20—N2—C19	116.7 (3)	C12—C11—C1	123.5 (3)
C20—N3—C21	109.1 (3)	C17—C12—C13	117.9 (3)
C23—N4—C21	105.4 (3)	C17—C12—C11	125.8 (3)
C23—N4—C24	129.4 (3)	C13—C12—C11	116.3 (3)
C21—N4—C24	125.1 (3)	C14—C13—C12	120.5 (4)
C23—N5—C22	104.1 (3)	C14—C13—H13A	119.7
C11—C1—C2	109.4 (3)	C12—C13—H13A	119.7
C11—C1—C8	112.4 (3)	C13—C14—C15	121.7 (4)
C2—C1—C8	107.7 (3)	C13—C14—H14A	119.2
C11—C1—C9	110.0 (3)	C15—C14—H14A	119.2
C2—C1—C9	107.3 (3)	C14—C15—C16	118.1 (4)
C8—C1—C9	109.9 (3)	C14—C15—C18	120.7 (3)
C1—C2—C3	111.8 (3)	C16—C15—C18	121.2 (3)
C1—C2—H2B	109.3	C15—C16—C17	121.1 (4)
C3—C2—H2B	109.3	C15—C16—H16A	119.4
C1—C2—H2C	109.3	C17—C16—H16A	119.4
C3—C2—H2C	109.3	C12—C17—C16	120.7 (4)
H2B—C2—H2C	107.9	C12—C17—H17A	119.7
C4—C3—C2	109.6 (3)	C16—C17—H17A	119.7
C4—C3—C10	109.1 (3)	N1—C18—C15	114.4 (3)
C2—C3—C10	108.9 (3)	N1—C18—H18A	108.7
C4—C3—H3B	109.8	C15—C18—H18A	108.7
C2—C3—H3B	109.8	N1—C18—H18B	108.7
C10—C3—H3B	109.8	C15—C18—H18B	108.7
C3—C4—C5	109.6 (3)	H18A—C18—H18B	107.6
C3—C4—H4A	109.8	N1—C19—N2	118.8 (3)
C5—C4—H4A	109.8	N1—C19—C22	122.5 (3)
C3—C4—H4B	109.8	N2—C19—C22	118.8 (3)
C5—C4—H4B	109.8	N3—C20—N2	131.9 (3)
H4A—C4—H4B	108.2	N3—C20—Cl1	114.8 (3)
C6—C5—C9	110.0 (3)	N2—C20—Cl1	113.3 (3)
C6—C5—C4	109.3 (3)	N3—C21—N4	126.1 (3)
C9—C5—C4	109.2 (3)	N3—C21—C22	127.8 (3)
C6—C5—H5B	109.4	N4—C21—C22	106.1 (3)
C9—C5—H5B	109.4	N5—C22—C21	110.4 (3)
C4—C5—H5B	109.4	N5—C22—C19	133.8 (3)
C5—C6—C7	110.1 (3)	C21—C22—C19	115.7 (3)
C5—C6—H6A	109.6	N5—C23—N4	113.9 (3)
C7—C6—H6A	109.6	N5—C23—H23A	123.0
C5—C6—H6B	109.6	N4—C23—H23A	123.0
C7—C6—H6B	109.6	N4—C24—C26	110.1 (3)
H6A—C6—H6B	108.2	N4—C24—C25	109.2 (3)
C10—C7—C8	109.2 (3)	C26—C24—C25	113.3 (4)
C10—C7—C6	109.4 (3)	N4—C24—H24A	108.0
C8—C7—C6	110.0 (3)	C26—C24—H24A	108.0
C10—C7—H7A	109.4	C25—C24—H24A	108.0
C8—C7—H7A	109.4	C24—C25—H25A	109.5
C6—C7—H7A	109.4	C24—C25—H25B	109.5
C7—C8—C1	110.4 (3)	H25A—C25—H25B	109.5
C7—C8—H8A	109.6	C24—C25—H25C	109.5
C1—C8—H8A	109.6	H25A—C25—H25C	109.5
C7—C8—H8B	109.6	H25B—C25—H25C	109.5
C1—C8—H8B	109.6	C24—C26—H26A	109.5
H8A—C8—H8B	108.1	C24—C26—H26B	109.5
C5—C9—C1	109.6 (3)	H26A—C26—H26B	109.5
C5—C9—H9A	109.7	C24—C26—H26C	109.5
C1—C9—H9A	109.7	H26A—C26—H26C	109.5
C5—C9—H9B	109.7	H26B—C26—H26C	109.5
C1—C9—H9B	109.7	Cl11—C27—Cl13	110.5 (3)
H9A—C9—H9B	108.2	Cl11—C27—Cl12	109.7 (3)
C7—C10—C3	109.4 (3)	Cl13—C27—Cl12	110.1 (3)
C7—C10—H10A	109.8	Cl11—C27—H27A	108.8
C3—C10—H10A	109.8	Cl13—C27—H27A	108.8
C7—C10—H10B	109.8	Cl12—C27—H27A	108.8
C3—C10—H10B	109.8		

### Hydrogen-bond geometry (Å, °)

**Table:**

*D*—H···*A*	*D*—H	H···*A*	*D*···*A*	*D*—H···*A*
N1—H1A···N5^i^	0.88	2.22	3.013 (5)	150
C27—H27A···N2^ii^	1.00	2.59	3.553 (6)	161
C5—H5B···N3^iii^	1.00	2.66	3.641 (5)	166
C23—H23A···O1^iv^	0.95	2.23	3.179 (5)	175

Symmetry codes: (i) −*x*, −*y*+1, −*z*+1; (ii) −*x*+1/2, −*y*+2, *z*+1/2; (iii) *x*, −*y*+3/2, *z*+1/2; (iv) *x*, *y*−1, *z*.
